# Redefine the role of range shifter in treating bilateral head and neck cancer in the era of Intensity Modulated Proton Therapy

**DOI:** 10.1002/acm2.12416

**Published:** 2018-07-16

**Authors:** Xuanfeng Ding, Xiaoqiang Li, An Qin, Jun Zhou, Di Yan, Peter Chen, Chinnaiyan Prakash, Craig Stevens, Rohan Deraniyagala, Peyman Kabolizadeh

**Affiliations:** ^1^ Department of Radiation Oncology Beaumont Health Royal Oak Michigan

## 1 INTRODUCTION

The use of proton beam therapy has been increasing rapidly. As the Pencil Beam Scanning (PBS) technology has become commercially available in the recent 10 years,^1^ nearly all the new proton centers under the contract or constructions are now configured with only PBS technique. Compared to passive‐scattering technique, Intensity Modulated Proton Therapy (IMPT) based on PBS technique allows for creating a more conformal dose distribution to target volume while resulting in a less body integral dose and ultimately less neutron dose.^1^, ^2^ For a majority of the current commercial proton beam systems, the minimum proton beam energy ranges from 70 to 100 MeV which is about 4.1–7.5 cm in water‐equivalent thickness (WET). In order to treat superficial target volume such as patients with head and neck cancer (HNC), and brain tumors, a range shifter (RS) is normally needed to attenuate the proton beam energy.^3^, ^4^, ^5^

The RS which is normally composed of slab of plastics such as acrylonitrile butadiene styrene (ABS) and polyethylene 3, 4, 5 which broadens the proton beams due to the secondary scattering.^6^ In order to reduce the scattering and keep a smaller spot size, the air gap between the gantry nozzle and patient's skin must be reduced. Thus, the proton gantry nozzle causes potential collision concerns with the patient's body especially in the vicinity of the shoulder region during the treatment of HNC.^7^ In order to avoid using RS, Both et al. introduced a rigid U‐shaped bolus placed close to the patient's head and neck region as an alternative.^4^ The advantage was to reduce the air gap and therefore the proton beam was able to maintain the spot size. However, as a trade‐off, it also introduced additional workload for the therapists to mount this heavy U‐shaped bolus on the table every time during the Computed Tomography (CT) simulation as well as to the couch following the daily imaging alignment prior to the radiation delivery. Another challenge is the size of bolus which needs to be carefully selected to fit patient's anatomy. Additionally, it also introduces WET inhomogeneity at the edge of bolus near the connection area to the couch top which limits the proton beam angle selections due to the range uncertainties.^4^

It is very difficult to model a continuous moving RS configuration due to the secondary proton scattering. Shen et al.^8^ and Li et al.^9^ have been addressing these issues using analytical model as well as an in‐house Monte Carlos simulation. Moreover, commissioning of a RS also requires extensive measurements.^8^ To overcome such limitations in proton beam therapy, air gap <10 cm between the patient body and RS is recommended in order to minimize the dose calculation error.^10^ In addition, larger air gap results in a larger spot size which makes robust coverage of the CTV while sparing critical structures less likely. Thus, to minimize such air gap, the potential collisions between the gantry nozzle and patient's shoulder became a concern in treating bilateral HNC. With so many disadvantages and inconvenient clinical workflow with using RS, it is critical to evaluate the role of RS and possibly eliminating it in treating bilateral HNC patient using IMPT.

Normally, three to four field IMPT with RS were used in the bilateral HNC treatment. We hypothesize that by increasing the degree of freedom or beam angle directions, IMPT is able to deliver a robust prescription dose to the bilateral HNC target without using RS. Such IMPT planning technique could effectively reduce the secondary proton scatter from RS which reduces the spot size entering the patient surface. This IMPT planning approach without RS would improve the dosimetric outcome in treatment bilateral HNC over the traditional planning approach using RS. To the best of our knowledge, this is the first planning study to explore such possibility by using different arrangement and number of proton beam fields.

## 2 MATERIALS AND METHODS

### 2.A Plan objective function vs. number of beam angles

Ten bilateral HNC cases were randomly selected from the patient population who received photon treatment in our clinic in the last 2 years to quantitatively evaluate the “plan quality” with RS or without RS. A series of IMPT plans with RS (IMPT_RS) and without RS (IMPT_noRS) were planned on the same CT scan structure set while increasing the number of beam angle from 3 to 10. RayStation version 5.02 (RaySearch Laboratories AB, Stockholm, Sweden) was used in this series of dosimetric studies. Robust optimization parameters were set to ±3.5% range and ±3 mm in x,y,z directions. Clinical Target Volume (CTV) was prescribed to 70 Gy[RBE] to high risk volume and 60 Gy[RBE] to intermediate/low risk volume. Air gap of IMPT_RS plans were set at 10 cm to ensure both spot size and clearance between the RS and patient body. The plan optimization parameters were set to 60 interactions with 0.02 minimum spot Monitor Unit (MU). For a fair comparison, all plans in the IMPT_RS and IMPT_noRS groups used the same beam angles and objective functions for the target volume and the organs at risks (OARs). The final average plan objective values were analyzed which represent the deviation from the plan objective function.^11^ In some proton nozzle designs, it might be difficult to reach 10 cm air gap. In the supplemental document, we also included a RS planning group with 15 cm air gap (IMPT_RS_15 cm).

### 2.B A comprehensive plan quality comparison study: four‐field IMPT with RS vs. four‐field IMPT without RS

To find out whether the plan quality could be improved via IMPT_noRS, a series of four‐field IMPT with RS (4F IMPT_RS) and without RS (4F IMPT_noRS) plans were generated. Dosimetric quality of each plan was evaluated via organ‐at‐risks (OARs) dose volume histogram, target conformity (CI) and target volume homogeneity indexes (HI) based on the Radiation Therapy Oncology Group (RTOG) recommendations. The plan robustness was evaluated using the root‐mean‐square deviation doses (RMSDs).^12^ In this study, RMSDs of the 21 scenarios (with ±3.5% range uncertainties, and setup uncertainties of ±3 mm for x,y,z directions) were calculated for every voxel. The area under the RMSD volume histograms (RVHs) curve (AUC) indicates the plan robustness.^12^ This concept is to quantitatively evaluate the plan robustness in the presence of setup and range uncertainties. It calculated dose difference in each voxel in these worst‐case‐scenarios. The smaller the AUC value was, the more robust the plan for the corresponding structure was. Patient geometry change is not included in this calculation. Treatment beam delivery time was estimated based on a 360 degree gantry room with 1 RPM gantry rotation speed, 2 ms spot switching time and a energy‐layer‐switching‐time (ELST) from 0.1 to 5 seconds.^13^ Statistical analysis was evaluated with two‐sided paired t‐test.

## 3 RESULTS

### 3.A Plan objective function vs. number of beam angles

**Figure 1:**
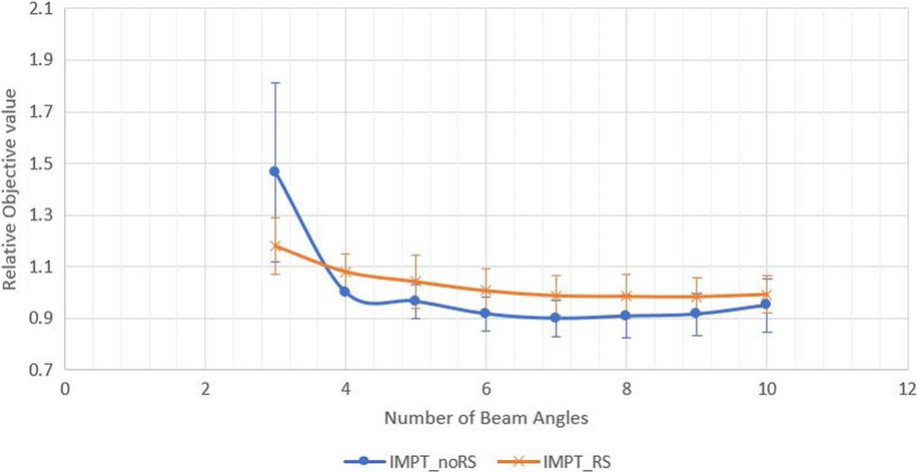
The average plan objective value vs. number of beam angles with using RS IMPT_RS (blue) or without RS IMPT_noRS (red) of ten patients. The relative objective value is normalized to 4F IMPT_noRS plan

The final average plan objective values of 10 cases were plotted as a function of number beam angles with both IMPT_RS and IMPT_noRS series (Fig. 1). Higher objective value indicates the plan deviate more from the same objective functions. This result showed a very interesting phenomenon that IMPT_noRS plan objective value was actually lower than IMPT_RS when the beam number was increased to four or more. In other words, it is not necessary to use RS in treating bilateral HNC via IMPT if four or more beam angles are used and the plan quality could be further improved. Our results did however confirm that RS is highly needed and recommended for treatment of bilateral HNC if <4 fields are used in IMPT robust planning (Fig. 1).

### 3.B Plan quality comparison: four‐field IMPT with RS vs. four‐field IMPT without RS

**Figure 2:**
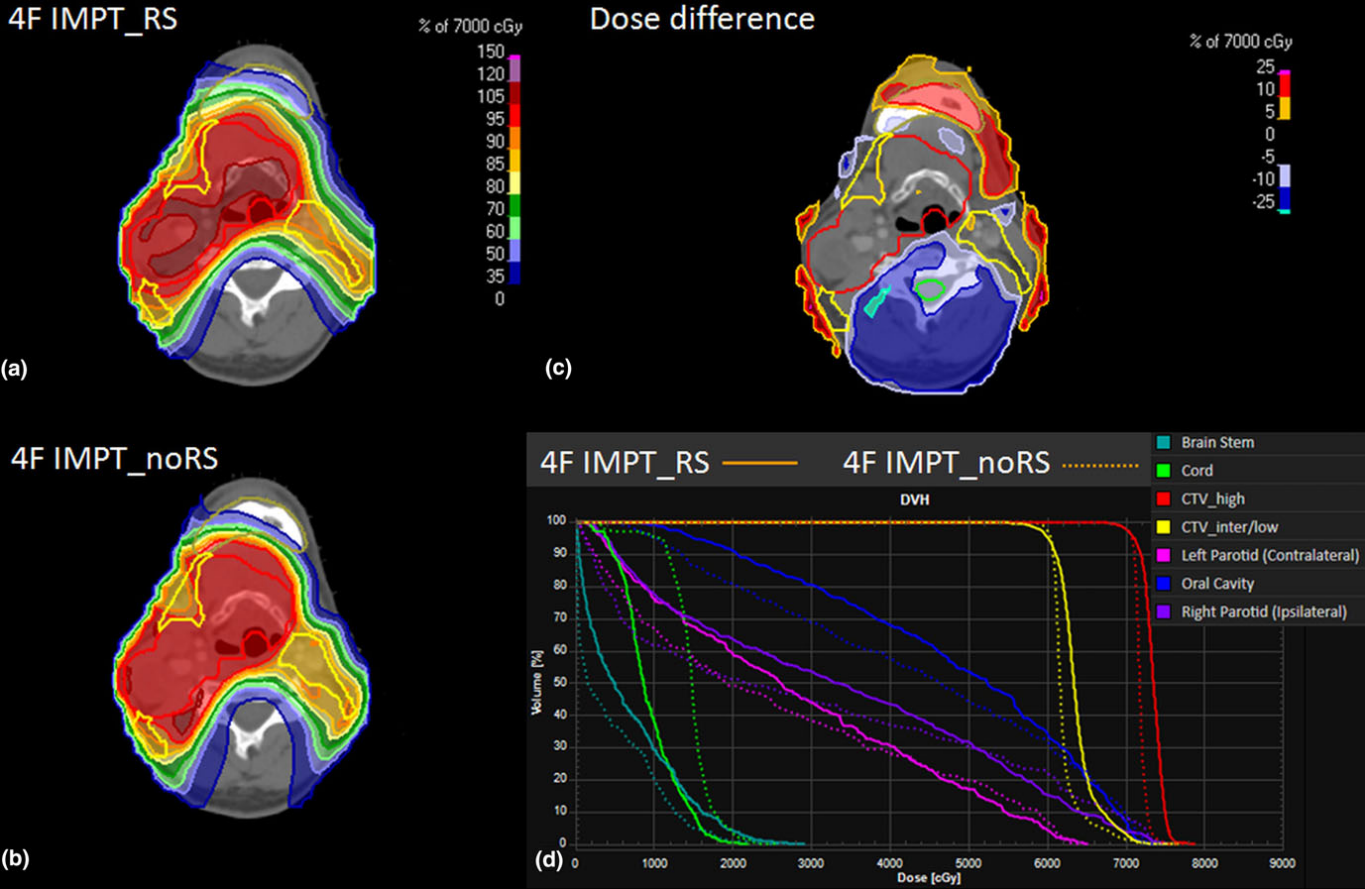
A representative CT slice and dose distribution between 4F IMPT_RS (A) and 4F IMPT_noRS (B). Dose difference: (4F IMPT_RS subtracts 4F IMPT_noRS plan); (C) and DVH's comparison (D). Both plans used the same beam angle and same objective function.

**Figure 3:**
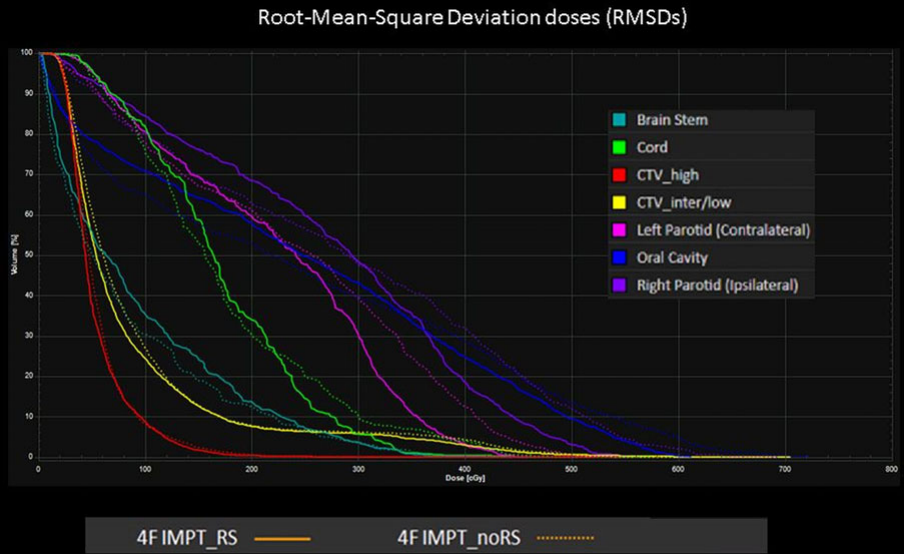
A representative case of root‐mean‐square deviation dose comparison between 4F IMPT_RS and 4F IMPT_noRS

The initial result shows that 4F IMPT_noRS is able to provide an overall equivalent or better plan quality over 4F IMPT_RS in terms of target coverage and OARs sparing (Fig. 2) in which CTV_high_ and CTV_inter/low_ HI was improved with an average of 0.95 to 0.97 (*p *=* *0.007) and of 0.96 to 0.97 (*p* = 0.004) respectively (Table 1). Mean dose to ipsilateral parotid gland and skin was reduced from 34.1 Gy [RBE] to 32.10 Gy [RBE] (*p *=* *0.007) and 16.26 [RBE] to 11.99 Gy [RBE] (*p *=* *0.031) respectively. Furthermore, root‐mean square deviation dose comparison between 4F IMPT_RS and 4F IMPT_noRS was plotted in Fig. 3. The AUC calculation indicates that 4F IMPT_noRS is able to provide a comparable or better robustness over 4F IMPT_RS in the target and organs at risk with an exception of skin (Table 1). As a trade‐off, 4F IMPT_noRS requires additional average of 7.5 more energy layers per plan due to the smaller spot size and narrower pristine bragg peak without using RS (*p *=* *0.001). As a result, the beam delivery time using 4F IMPT_noRS was slightly longer than 4F IMPT_RS (Table 1).

**Table 1:** Dosimetric and plan parameter comparison of 4F IMPT_RS and 4F IMPT_noRS of ten patients

Structures	Value	4F IMPT_RS	4F IMPT_noRS	Relative difference to 4F IMPT_RS
				4F IMPT_noRS
CTV_high_	D98%(Gy [RBE])	70	70	–
HI of CTV_high_		0.95 ± 0.02	0.97 ± 0.01	+0.02 (*p *=* *0.007)
CI of CTV_high_		0.54 ± 0.11	0.52 ± 0.09	−0.02 (*p *=* *0.332)
	AUC	67.80 ± 17.16	65.70 ± 9.66	−2.10 (*p *=* *0.640)
HI of CTV_inter/low_		0.96 ± 0.02	0.97 ± 0.02	+0.01 (*p *=* *0.004)
	AUC	69.70 ± 14.43	74.30 ± 10.99	+4.6 (*p *=* *0.139)
Ipsilateral Parotid	Mean(Gy [RBE])	34.10 ± 6.07	32.10 ± 6.49	−2.00 (*p *=* *0.007)
	AUC	296.00 ± 52.64	317.70 ± 31.39	+21.70 (*p *=* *0.097)
Contralateral Parotid	Mean(Gy [RBE])	27.43 ± 2.16	24.99 ± 7.51	−2.44 (*p *=* *0.263)
	AUC	258.70 ± 18.57	279.80 ± 30.49	+21.10 (*p *=* *0.090)
Ipsilateral Submandibular Gland	Mean(Gy [RBE])	68.38 ± 2.34	66.79 ± 2.74	−0.41 (*p *=* *0.068)
	AUC	148.40 ± 78.48	166.30 ± 78.80	+17.90 (*p *=* *0.436)
Contralateral Submandibular Gland	Mean(Gy [RBE])	52.70 ± 5.91	53.29 ± 7.30	+0.59 (*p *=* *0.645)
	AUC	268.90 ± 82.07	294.40 ± 90.93	+25.50 (*p *=* *0.238)
Spinal Cord	Max (Gy[RBE])	25.20 ± 6.02	27.52 ± 7.78	+2.32 (*p *=* *0.087)
	AUC	99.60 ± 28.57	97.60 ± 24.15	−2.00 (*p *=* *0.782)
Brainstem	Max (Gy[RBE])	23.71 ± 8.06	22.00 ± 8.24	−1.71 (*p *=* *0.053)
	Mean(Gy [RBE])	5.78 ± 2.84	5.06 ± 3.26	−0.72 (*p *=* *0.097)
	AUC	93.80 ± 33.54	78.50 ± 36.53	−15.30 (*p *=* *0.001)
Mandible	Mean(Gy [RBE])	29.22 ± 6.80	27.37 ± 8.40	−1.85 (*p *=* *0.090)
	Max (Gy[RBE])	73.01 ± 5.06	72.55 ± 5.01	−0.46 (*p *=* *0.335)
	AUC	246.40 ± 51.22	262.50 ± 60.31	+16.10 (*p *=* *0.107)
Oral Cavity	Mean(Gy [RBE])	26.37 ± 11.74	24.77 ± 12.66	−1.60 (*p *=* *0.062)
	AUC	278.80 ± 69.95	288.00 ± 73.65	+9.20 (*p *=* *0.107)
Skin	Max (Gy[RBE])	67.13 ± 4.28	64.20 ± 7.94	−2.93 (*p *=* *0.089)
	Mean(Gy [RBE])	16.26 ± 5.81	11.99 ± 3.26	−4.27 (*p *=* *0.031)
	AUC	102.00 ± 27.68	120.80 ± 18.81	+18.80 (*p *=* *0.040)
Body integral dose	Relative Number	1	0.94 ± 0.10	−6% (*p *=* *0.047)
Total Energy Layers		142.80 ± 9.67	150.30 ± 7.50	+7.50 (*p *=* *0.001)
Total Monitor Unit		2367.48 ± 391.51	2247.11 ± 356.70	−120.37 (*p *=* *0.001)
Average energy layers per control points		35.70 ± 2.42	37.58 ± 1.87	+1.88 (*p *=* *0.001)
Total Delivery time (ELST* *=* *5s)	(seconds)	976 ± 96	1040 ± 75	+64 (*p *<* *0.001)
Total Delivery Time (ELST* *=* *4s)	(seconds)	835 ± 87	891 ± 70	+56.10 (*p *<* *0.001)
Total Delivery Time (ELST* *=* *3s)	(seconds)	694 ± 79	742 ± 65	+48 (*p *<* *0.001)
Total Delivery Time (ELST* *=* *2s)	(seconds)	552 ± 71	592 ± 60	+40 (*p *=* *0.001)
Total Delivery Time (ELST* *=* *1s)	(seconds)	411 ± 63	443 ± 56	+32 (*p *=* *0.003)
Total Delivery Time (ELST* *=* *0.5s)	(seconds)	340 ± 59	368 ± 55	+28 (*p *=* *0.010)
Total Delivery Time (ELST* *=* *0.2s)	(seconds)	298 ± 57	324 ± 54	+26 (*p *=* *0.015)
Total Delivery Time (ELST* *=* *0.1s)	(seconds)	284 ± 56	309 ± 54	+25 (*p *=* *0.017)

## 4 DISCUSSION

**Figure 4:**
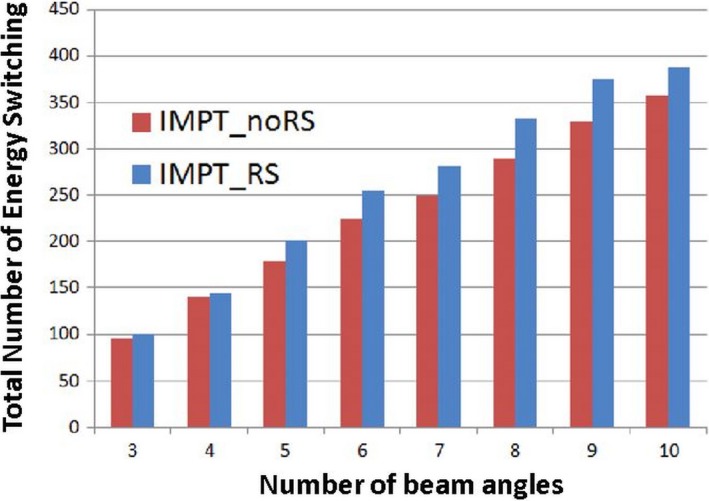
Total number of energy layers switching as a function of number of beam angles from a representative case. IMPT_noRS (blue) usually contains less total energy layers compared to the IMPT_RS (red).

Based on the data presented, it is concluded that RS may not be needed to treat bilateral HNC case via IMPT robust optimization with four or more beam angles. Moreover, the study finds that plan objective value will continue to drop as the number of beam angles is increasing (Fig. 1). Hence, a better plan quality can be achieved with more beam angles. However, the delivery time also prolongs as the number of energy layers and spots increases with more beam angles used (Fig. 4) which result in a longer delivery time per plan. In current routine clinical practice, the maximum number of proton beam angles used for treating bilateral HNC patients is four fields. As the ELST is still the most important concept for the PBS delivery efficiency, there has been multiple publications 14, 15 on the energy layer reduction methods to shorten the beam delivery time for IMPT and results has been promising. Thus, the most important question is to evaluate the feasibility of using more beam angles while reducing the delivery time while maintaining similar plan quality.

### 4.A Explore the plan quality and feasibility of increasing the number of beam angles

**Figure 5:**
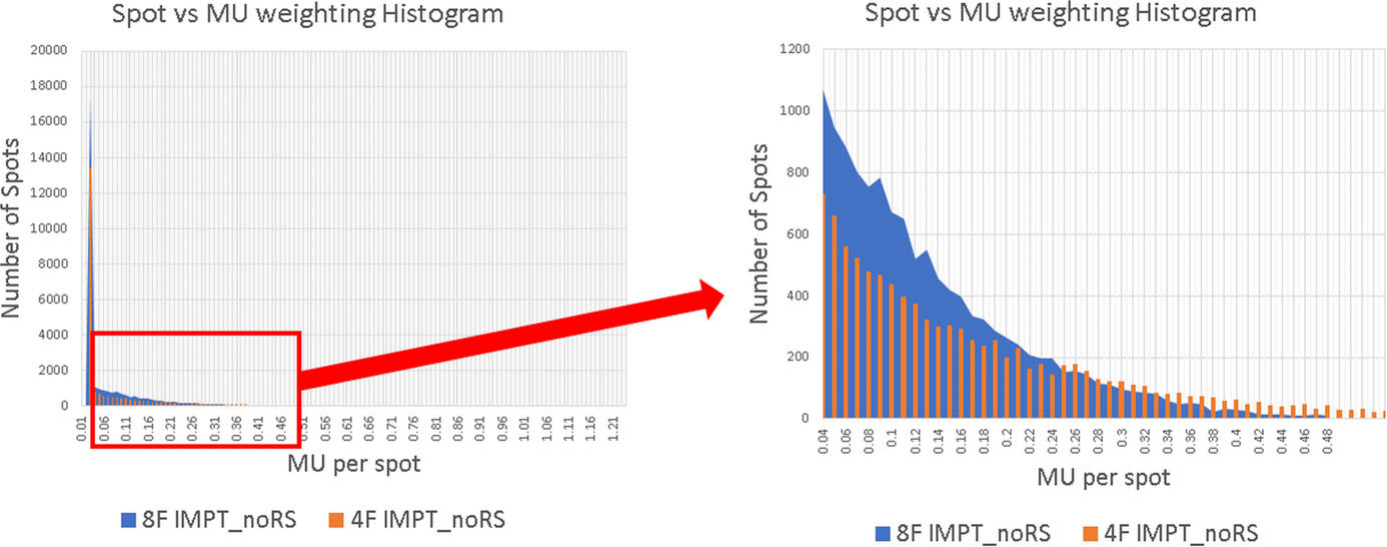
A representative case of spot vs MU weighting histogram analysis comparing 4F IMPT_noRS with 8F IMPT_noRS. Compared to the four‐field IMPT_noRS plan, the 8F IMPT_noRS contains much more spot which is <0.25MU.

To test the feasibility and the plan quality of using more beam angles for bilateral HNC patients, eight‐field IMPT without RS plans (8F IMPT_noRS) were created using the same 10 patient CT structure sets as described above. Beam angles in 8F IMPT_noRS were evenly distributed (45^o^ apart) and energy layer reduction method 14, 15 was used to reduce the total number of energy layers while maintaining a similar robust plan quality. Dosimetric quality and beam delivery time of each plan was evaluated by comparing 8F IMPT_noRS and 4F IMPT_noRS plan groups (Table 2). The results showed 8F IMPT_noRS was able to further reduce some of the OARs dose over 4F IMPT_noRS such as mean dose to ipsilateral parotid gland (32.10 Gy [RBE] to 30.27 Gy [RBE] *p* = 0.017); contralateral submandibular glands (53.29 Gy [RBE] vs 47.27 Gy [RBE] *p *=* *0.005); oral cavity (24.77 Gy [RBE] vs 21.97 Gy [RBE] *p* = 0.007), respectively. Plans’ robustness was comparable between the two groups. With the energy reduction method, beam‐on time of 8F IMPT_noRS could be less than 4F IMPT_noRS without energy reductions (*p *<* *0.005) (Table 2 and supplemental document Fig. 2). It was found that the treatment plan tended to use lower MU weighting per spots as the number of beam angle increased. Such phenomena will push the proton system to its delivery limitation. Fig. 5 shows spot weighting distribution of both 4F IMPT_noRS and 8F IMPT_noRS plans. For different proton machine with different minimum MU threshold, special care is need when more fields are used in clinic.

**Table 2:** Dosimetric and plan parameter comparison of 4F IMPT_noRS and 8F IMPT_noRS of ten patients

Structures	Value	4F IMPT_noRS	8F IMPT_noRS	Relative difference to 4F IMPT_noRS
				8F IMPT_noRS
CTV_high_	D98%(Gy [RBE])	70	70	–
HI of CTV_high_		0.97 ± 0.01	0.97 ± 0.01	0.00 (*p *=* *0.015)
CI of CTV_high_		0.52 ± 0.09	0.53 ± 0.10	+0.01 (*p *=* *0.531)
	AUC	65.70 ± 9.66	68.00 ± 9.06	+2.30 (*p *=* *0.070)
HI of CTV_inter/low_		0.97 ± 0.02	0.97 ± 0.02	0.00 (*p *=* *0.443)
	AUC	74.30 ± 10.99	77.80 ± 11.06	+3.50 (*p *=* *0.506)
Ipsilateral Parotid	Mean(Gy [RBE])	32.10 ± 6.49	30.27 ± 7.23	−1.83 (*p *=* *0.017)
	AUC	317.70 ± 31.39	318.9 ± 56.25	+1.20 (*p *=* *0.923)
Contralateral Parotid	Mean(Gy [RBE])	24.99 ± 7.51	22.93 ± 2.37	−2.06 (*p *=* *0.449)
	AUC	279.80 ± 30.49	276.50 ± 38.06	−3.30 (*p *=* *0.715)
Ipsilateral Submandibular Gland	Mean(Gy [RBE])	66.79 ± 2.74	66.28 ± 2.25	−0.51 (*p *=* *0.588)
	AUC	166.30 ± 78.80	166.40 ± 83.42	+0.10 (*p *=* *0.996)
Contralateral Submandibular Gland	Mean(Gy [RBE])	53.29 ± 7.30	47.27 ± 7.95	−6.02 (*p *=* *0.005)
	AUC	294.40 ± 90.93	321.50 ± 113.10	+27.10 (*p *=* *0.265)
Spinal Cord	Max (Gy[RBE])	27.52 ± 7.78	24.88 ± 4.58	−2.65 (*p *=* *0.157)
	AUC	97.60 ± 24.15	104.20 ± 26.89	+6.60 (*p *=* *0.462)
Brainstem	Max (Gy[RBE])	22.00 ± 8.24	20.14 ± 7.88	−1.86 (*p *=* *0.075)
	Mean(Gy [RBE])	5.06 ± 3.26	4.57 ± 3.13	−0.49 (*p *=* *0.086)
	AUC	78.50 ± 36.53	70.10 ± 32.14	−8.40 (*p *=* *0.055)
Mandible	Mean(Gy [RBE])	27.37 ± 8.40	28.34 ± 7.52	+0.97 (*p *=* *0.370)
	Max (Gy[RBE])	72.55 ± 5.01	73.25 ± 4.74	+0.70 (*p *=* *0.143)
	AUC	262.50 ± 60.31	244.10 ± 53.30	−18.40 (*p *=* *0.035)
Oral Cavity	Mean(Gy [RBE])	24.77 ± 12.66	21.97 ± 11.97	−2.81 (*p *=* *0.007)
	AUC	288.00 ± 73.65	300.60 ± 91.07	+12.60 (*p *=* *0.20)
Skin	Max (Gy[RBE])	64.20 ± 7.94	63.85 ± 6.94	−0.335 (*p *=* *0.489)
	Mean(Gy [RBE])	11.99 ± 3.26	13.46 ± 5.75	+1.47 (*p *=* *0.405)
	AUC	120.80 ± 18.81	112.50 ± 19.78	−8.30 (*p *=* *0.036)
Body integral dose	Relative Number	1	0.97 ± 0.07	−3% (*p *=* *0.364)
Total Energy Layers		150.30 ± 7.50	130.50 ± 19.52	−19.80 (*p *=* *0.009)
Total Monitor Unit		2247.11 ± 356.70	2125.12 ± 656.62	−121.99 (*p *=* *0.585)
Average energy layers per control points		37.58 ± 1.87	16.31 ± 2.44	−21.26 (*p *<* *0.001)
Total Delivery time (ELST* *=* *5s)	(seconds)	1040 ± 75	923 ± 109	−117 (*p *=* *0.002)
Total Delivery Time (ELST* *=* *4s)	(seconds)	891 ± 70	793 ± 91	−98 (*p *=* *0.002)
Total Delivery Time (ELST* *=* *3s)	(seconds)	742 ± 65	663 ± 73	−79 (*p *=* *0.001)
Total Delivery Time (ELST* *=* *2s)	(seconds)	592 ± 60	531 ± 58	−61 (*p *<* *0.001)
Total Delivery Time (ELST* *=* *1s)	(seconds)	443 ± 56	401 ± 46	−42 (*p *<* *0.001)
Total Delivery Time (ELST* *=* *0.5s)	(seconds)	368 ± 55	336 ± 42	−33 (*p *=* *0.002)
Total Delivery Time (ELST* *=* *0.2s)	(seconds)	324 ± 54	296 ± 40	−27 (*p *=* *0.003)
Total Delivery Time (ELST* *=* *0.1s)	(seconds)	309 ± 54	283 ± 40	−25 (*p *=* *0.005)

The proton therapy has become more utilized in the last decade and advances in techniques are in progress. Range shifters can cause many limitations in delivering proton radiotherapy and therefore, efforts are warranted to decrease the use of such device. In treating bilateral HNC patients, RS can be omitted if four or more beams angles are used. Dosimetric outcome could ultimately be better without using RS as there would be less scatter radiation due eliminating interactions with the RS materials. Using more beam angles also resulted in lower objective value and potential to improve the plan quality (Fig. 1). Delivering radiation with more beam angles however, could result in extending the delivery time. Therefore, the energy layer reduction methods could effectively reduced the beam delivery time making the 8F IMPT_noRS clinically feasible. Nevertheless, one of the limitation of this study is that the beam delivery time is estimated based on a single room system, as a result the room switching time and waiting time from a multiroom proton center were not estimated. Hence, additional time is needed to be added to compensate the difference between the single room and multiroom system system.^16^ In a busy multiroom center, IMPT with eight beam angles might still not be practically feasible due to the long waiting time. However, with the recent invention of Spot‐Scanning Proton Arc (SPArc) therapy,^13^, ^17^, ^18^ future proton therapy systems might be able to deliver a dynamic proton arc treatment via a single continuous arc rotation which could ensure both robust plan quality and delivery efficiency. Of course, there are still a lot of challenges in both gantry hardware and control system software in order to deliver such dynamic proton arc treatment in clinical settings.

## CONCLUSION

Through this study, it is warranted that each institution is able to find an optimum solution to compensate the plan robust quality as well as the clinical workflow and feasibility to treat bilateral HNC patients. Similar concept using IMPT without RS could be potentially applied to other disease sites with the target volume extended to the superficial area. However, each clinical case is different which may need fewer beams as possible to capitalize on the potential to remove dose from noneffected tissues.

## CONFLICT OF INTEREST

Xuanfeng Ding, Xiaoqiang Li and Di Yan have a pending patent based on the Spot‐scanning proton arc (SPArc) algorithm.

## Supporting information


**Fig. S1.** Comparison of relative objective value vs number of beam angles using different planning strategies: IMPT_RS_10 cm, IMPT_RS_15 cm and IMPT_noRS in all ten HNC patients.
**Fig. S2** A representative CT slice and dose distribution between 4F IMPT_noRS (A) and 8F IMPT_noRS (B). Dose difference (4F IMPT_noRS subtracts 8F IMPT_noRS plan); (C) and DVH's comparison (D).
