# Supplementary material for: Redefine the role of range shifter in treating bilateral head and neck cancer in the era of Intensity Modulated Proton Therapy
Source: J Appl Clin Med Phys. 2018 Jul 16;19(5):749–55. doi: 10.1002/acm2.12416 (PMC6123136; doi:10.1002/acm2.12416)

Supplementary Figure 1: Comparison of Relative Objective Value vs Number of beam angles using different planning strategies: IMPT_RS_10cm, IMPT_RS_15cm and IMPT_noRS in all 10 HNC patients.

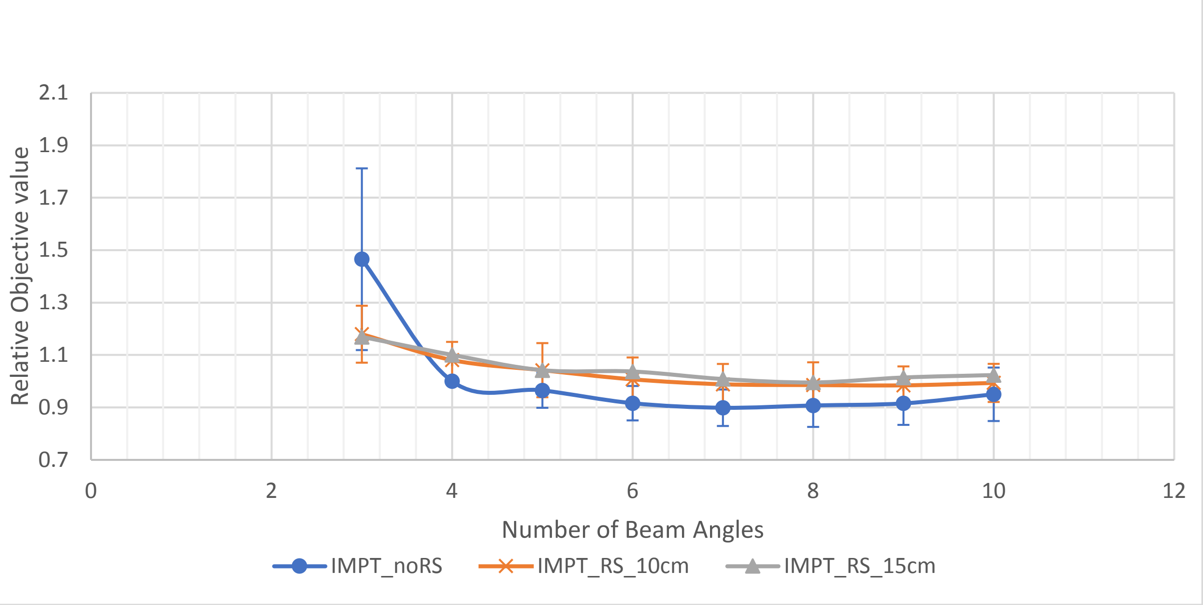

IMPT_noRS: Intensity Modulated Proton Therapy without using Range Shifter.
IMPT_RS_10cm: Intensity Modulated Proton Therapy using Range Shifter with air gap =10cm
IMPT_RS_15cm: Intensity Modulated Proton Therapy using Range Shifter with air gap =15cm

*The Relative Objective Value is normalized to 4F IMPT_noRS plan.

Supplementary Figure 2: A representative CT slice and dose distribution between 4F IMPT_noRS (A) and 8F IMPT_noRS (B). Dose difference (4F IMPT_noRS subtracts 8F IMPT_noRS plan); (C) and DVH's comparison (D)

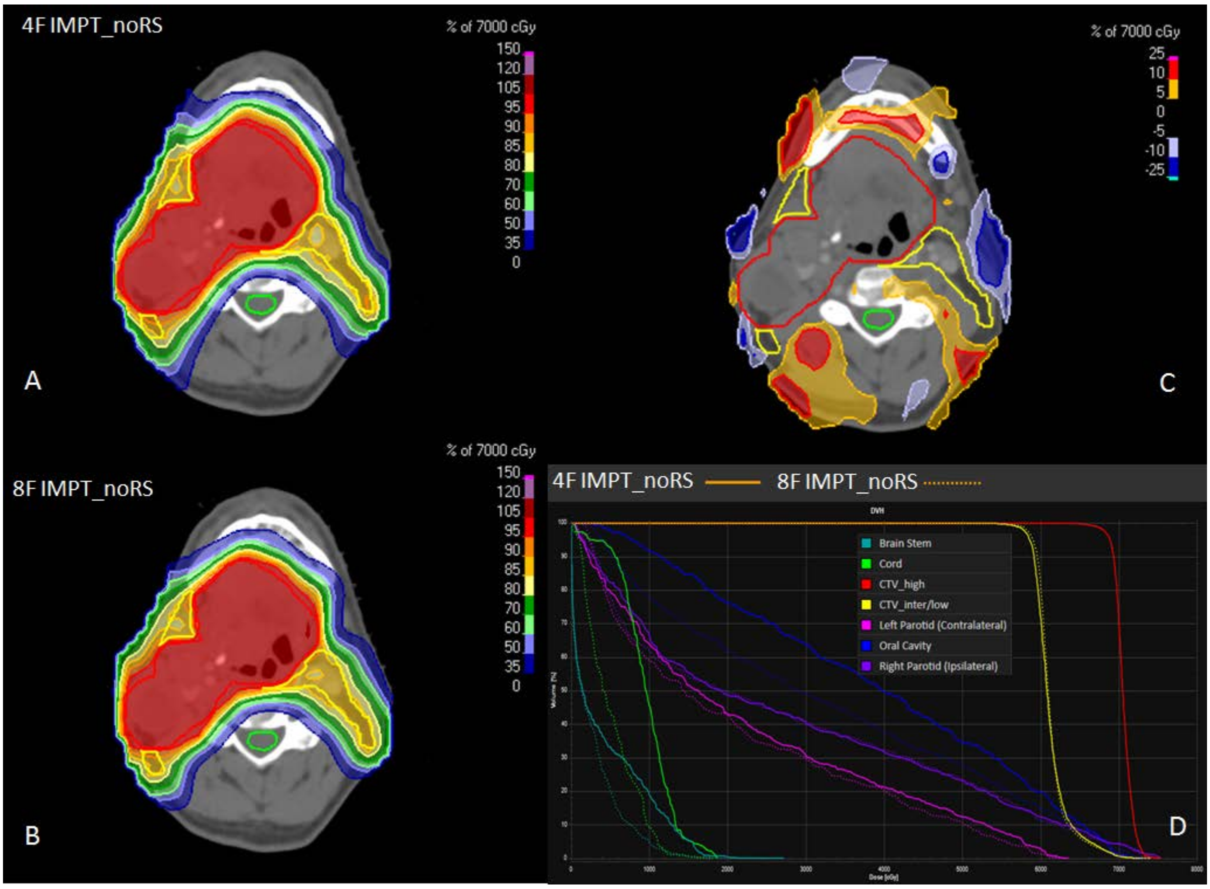

Supplement: Supplementary file 1 — Fig. S1. Comparison of relative objective value vs number of beam angles using different planning strategies: IMPT_RS_10 cm, IMPT_RS_15 cm and IMPT_noRS in all ten HNC patients. Fig. S2 A representative CT slice and dose distribution between 4F IMPT_noRS (A) and 8F IMPT_noRS (B). Dose difference (4F IMPT_noRS subtracts 8F IMPT_noRS plan); (C) and DVH's comparison (D). [file ACM2-19-749-s001.pdf]
